# Opportunities at the Interface of Network Science and Metabolic Modeling

**DOI:** 10.3389/fbioe.2020.591049

**Published:** 2021-01-25

**Authors:** Varshit Dusad, Denise Thiel, Mauricio Barahona, Hector C. Keun, Diego A. Oyarzún

**Affiliations:** ^1^Department of Life Sciences, Imperial College London, London, United Kingdom; ^2^Department of Mathematics, Imperial College London, London, United Kingdom; ^3^Department of Surgery and Cancer, Imperial College London, London, United Kingdom; ^4^Department of Metabolism, Digestion and Reproduction, Imperial College London, London, United Kingdom; ^5^School of Biological Sciences, University of Edinburgh, Edinburgh, United Kingdom; ^6^School of Informatics, University of Edinburgh, Edinburgh, United Kingdom

**Keywords:** genome scale metabolic modeling, network science, systems biology, flux balance analysis, machine learning, synthetic biology

## Abstract

Metabolism plays a central role in cell physiology because it provides the molecular machinery for growth. At the genome-scale, metabolism is made up of thousands of reactions interacting with one another. Untangling this complexity is key to understand how cells respond to genetic, environmental, or therapeutic perturbations. Here we discuss the roles of two complementary strategies for the analysis of genome-scale metabolic models: Flux Balance Analysis (FBA) and network science. While FBA estimates metabolic flux on the basis of an optimization principle, network approaches reveal emergent properties of the global metabolic connectivity. We highlight how the integration of both approaches promises to deliver insights on the structure and function of metabolic systems with wide-ranging implications in discovery science, precision medicine and industrial biotechnology.

## 1. Introduction

Metabolism comprises the biochemical reactions that convert nutrients into biomolecules and energy to sustain cellular functions. Advances in high-throughput screening technologies have enabled the quantitative characterization of metabolites, proteins and nucleic acids at the genome-scale, revealing previously unknown links between metabolism and many other cellular processes. For example, gene regulation (Chubukov et al., 2014), signal transduction (Tretter et al., 2016), immunity (Loftus and Finlay, 2016), and epigenetic modifications (Reid et al., 2017) have been shown to interact closely with metabolic processes. The increasing availability of data and the fundamental roles of metabolism in various cellular phenotypes (Tonn et al., 2019) have triggered a surge in metabolic research, together with a revived need for computational methods to untangle its complexity.

At the genome scale, metabolism comprises multiple interconnected reactions devoted to the production of energy and synthesis of essential biomolecules (e.g., proteins, lipids, or nucleic acids). The notion of a metabolic pathway is typically employed to organize sets of related reactions into functionally cohesive subsystems. Thus, lipid pathways, for example, are traditionally studied as distinct subsystems from amino acid or aerobic respiration pathways. Although conveniently descriptive, such *a priori* partitioning can obscure the links between other relevant layers of metabolic organization. Furthermore, metabolic connectivity is not static but actively responds and adapts to extracellular cues. Through various layers of transcriptional, translational, and post-translational regulation, metabolic pathways can be activated or shut down depending on external perturbations. These metabolic shifts drive a number of fundamental biological processes, such as microbial adaptations to growth conditions (Dai et al., 2016; Hartline et al., 2020) or the ability of pathogens to rewire their metabolism and evade the action of antimicrobial drugs (Olive and Sassetti, 2016). Metabolic adaptations are also thought to modulate the onset of complex diseases such as cancer (Hanahan and Weinberg, 2011; Pavlova and Thompson, 2016), diabetes, Alzheimer's, among others (DeBerardinis and Thompson, 2012; Suhre and Gieger, 2012). As a result, there is a growing need for computational tools that go beyond classical pathway definitions and can uncover hidden relations between metabolic components.

The complexity of metabolism has prompted the development of a myriad of methods to analyse its connectivity (Wishart et al., 2018). For specific pathways, kinetic models based on differential equations are widely employed to describe temporal dynamics of metabolites (Steuer et al., 2006; Saa and Nielsen, 2017). At the genome scale, however, the construction of kinetic models faces substantial challenges (Srinivasan et al., 2015). Such models require a large number of parameters, many of which have not been experimentally measured, or their values are subject to large uncertainty. As a result, the majority of genome-scale analyses are based on the metabolic stoichiometry alone. A widely adopted method for genome-scale modeling is Flux Balance Analysis (Palsson, 2015) (FBA), a powerful framework to predict metabolic fluxes on the basis of an optimization principle applied to the network stoichiometry. Alternatively, from the stoichiometry one can build graphs, a computational description of complex systems that has become the cornerstone of network science (Newman, 2010).

In this paper we discuss the relationship between FBA and graph-based analyses of metabolism, and we underline the complementary perspectives they bring to the understanding of metabolic organization. On the one hand, FBA has been shown to predict metabolic activity in various environmental and genetic contexts; on the other, network science can shed light on the emergent properties of global metabolic connectivity. Both approaches share a common root in the genome-scale stoichiometry of cellular metabolism, yet they offer different tools for its analysis. In the following, we discuss their advantages and caveats, highlighting the need and opportunities for integrated methods that combine flux optimization with network science.

## 2. Flux Balance Analysis

A large number of methods have been developed for the analysis of genome-scale metabolic networks (Lewis et al., 2012); these are generally described as *constraint-based methods* (Orth et al., 2010), an umbrella term for various techniques focused on the solution of the steady state equation:

(1) $$\begin{array}{l} \text{S}v=0, \end{array}$$

where **S** is the *n* × *m* stoichiometry matrix for a model with *n* metabolites and *m* reactions, and *v* is a vector containing the *m* reaction fluxes.

In general, Equation (1) is satisfied by an infinite number of flux vectors. A number of methods aim at probing the geometry of such flux solution space. For example, Elementary Flux Modes (Klamt et al., 2017) and Extreme Pathways (Wagner and Urbanczik, 2005) are two complementary techniques for decomposing the solution space into simpler units (Zanghellini et al., 2013; Muller and Bockmayr, 2014). Other methods for exploring the solution space include random flux sampling with Monte Carlo methods (Wiback et al., 2004), the use of dimensionality reduction techniques (Bhadra et al., 2018), and various structural decompositions of the stoichiometric matrix (Ghaderi et al., 2020).

The most widespread method for genome-scale modeling is Flux Balance Analysis (FBA), which selects a vector of metabolic fluxes *v* in Equation (1) as a solution to the optimization problem:

(2) $$\begin{array}{l} \;{\text{max}}_{v}J(v)\\ \text{subject to: }Sv=0\\ \;V_{i}^{\text{min}}\leq v_{i}\leq V_{i}^{\text{max}},\;i=1,\ldots ,m, \end{array}$$

where $\left(V_{i}^{\text{min}},V_{i}^{\text{max}}\right)$ are bounds on each flux. The objective function *J*(*v*) is chosen to describe the physiology of a particular organism under study. In microbes, biomass production is the most common choice for the objective function, in which *J*(*v*) = *c*^*T*^·*v*, i.e., the rate of biomass production is assumed to be a linear combination of specific biosynthetic fluxes, defined by the positive vector of weights *c*. There are many dedicated FBA software packages (Lakshmanan et al., 2014; Lieven et al., 2020) and its popularity has led to a myriad of extensions (Lewis et al., 2012) that account for other complexities of cell physiology such as gene regulation (Covert et al., 2008), dynamic adaptations (Rügen et al., 2015; Waldherr et al., 2015), and many others (Heirendt et al., 2019).

Flux Balance Analysis has found applications in diverse domains, including cell biology (McCloskey et al., 2013), metabolic engineering (Nielsen and Keasling, 2016), microbiome studies (Khandelwal et al., 2013; Manor et al., 2014; Rosario et al., 2018), and personalized medicine (Diener and Resendis-Antonio, 2016; Nielsen, 2017; Raškevičius et al., 2018). A salient feature of FBA is its ability to incorporate various types of omics datasets into its predictions. Various approaches have been developed for this purpose (Becker and Palsson, 2008; Colijn et al., 2009; Jerby et al., 2010; Lee et al., 2012a; Wang et al., 2012; Agren et al., 2014; Nam et al., 2014; Yizhak et al., 2014), most of which incorporate experimental data into the metabolic model through adjustments of the stoichiometric matrix **S** or the flux bounds $V_{i}^{\text{min}}$ and $V_{i}^{\text{max}}$ in (2).

A popular use case of FBA is the identification of essential genes, i.e., genes that severely impact cellular growth when knocked out. Through simulation of gene deletions, FBA can serve as a systematic tool for *in silico* screening of lethal mutations, and identification of biomarkers and drug targets in disease (Lehár et al., 2009; Raman and Chandra, 2009; Folger et al., 2011; Gatto et al., 2015; Krueger et al., 2016; Pagliarini et al., 2016; Robinson and Nielsen, 2017). A related application of FBA is the study of metabolic robustness. Since only a fraction of all metabolic reactions are essential in a given environment, knocking out non-essential reactions often has little effect on the phenotype. This is because many reactions have functional backups through other pathways, so as to preserve cellular function in face of perturbations. By providing insights into the reorganization of fluxes under different conditions, FBA can also help improve our understanding of robustness to gene knockouts (Blank et al., 2005; Deutscher et al., 2006; Larhlimi et al., 2011; Palsson, 2015; Ho and Zhang, 2016), gene mutations (Fong and Palsson, 2004), and different growth conditions (Ibarra et al., 2002).

One limitation of FBA is the crucial importance of the objective function to be optimized, which needs to be designed to represent cellular physiology. In microbes, a common choice is maximization of growth rate, but it is questionable whether this is a realistic cellular objective across organisms or in different growth conditions (Schuetz et al., 2007; Feist and Palsson, 2010; Garćıa Sánchez and Torres Sáez, 2014). Although the vast majority of FBA studies rely on the maximization of cellular growth, other objective functions have been proposed, including maximization of ATP production (Nam et al., 2014) and minimization of substrate uptake rate (Raman and Chandra, 2009).

## 3. Network Science in Metabolic Modeling

Network science represents complex systems as graphs where the nodes are the system components and the edges describe interactions between them. This general description provides a framework for modeling large, interconnected systems across many disciplines, including biology, sociology, economics, and others (Newman, 2010). Numerous works have analyzed metabolism under the lens of network science. Graph-theoretic concepts such as degree distributions and centrality measures (Jeong et al., 2000; Wagner and Fell, 2001; Ma and Zeng, 2003b) can reveal structural features of the connectivity of the overall system, while clustering algorithms can uncover substructures hidden in the network topology. Such tools can be combined with the analysis of perturbations, such as deletions of network nodes or edges (Palumbo et al., 2005; Larhlimi et al., 2011), which can represent changes in the environment, gene knockouts, or therapeutic drugs that target specific metabolic enzymes. Unlike FBA, in which the analysis depends on the choice of a specific objective function, network methods rely on the metabolic stoichiometry alone.

Metabolic modularity is an area where network science has shown promising results. Intuitively, a network module is a subset of the network containing nodes that are more connected among themselves than to the rest of the network. Several studies have focused on the modularity of metabolism, and how network modules can be used to coarse-grain the metabolic connectivity into subunits (Ma and Zeng, 2003b; Tanaka et al., 2005; Zhao et al., 2007; da Silva et al., 2008; Kreimer et al., 2008). The modules identified with network analysis have been found to capture the organization of textbook biochemical pathways while uncovering novel links and relationships between them (Ravasz et al., 2002). A recurring theme in these analyses is the bow-tie topology, whereby a metabolic network can be divided into an input component, an output component and a strongly connected internal component. This architecture aligns well with an intuitive understanding of metabolism, which comprises nutrient uptake, waste production and secretion, and a large number of internal cycles which produce biomass and energy (Ma and Zeng, 2003b; Tanaka et al., 2005; Zhao et al., 2007; da Silva et al., 2008; Kreimer et al., 2008; Cooper and Barahona, 2010).

Despite its promise, however, network science has generally achieved mixed success in metabolic research. For example, from a network perspective it would be natural to expect that essential genes should be associated with high centrality scores (Jeong et al., 2001; Plaimas et al., 2010; Raman et al., 2014; Jalili et al., 2016). This idea draws parallels from other domains, such as the internet and social networks, where highly central nodes are deemed critical for network connectivity. However, correlations between gene essentiality and node centrality have been so far shown to be weak, with various essential metabolites and reactions exhibiting low centrality scores (Mahadevan and Palsson, 2005; Samal et al., 2006). This happens because poorly connected nodes are often the sole route for producing precursors that are essential for growth; in other words, such nodes lack a functional backup that can compensate for their loss. For example, Samal et al. (2006) showed that more than 50% of essential reactions in *Escherichia coli, Saccharomyces cerevisiae*, and *Staphylococcus aureus* are involved in such unique pathways, while other works noted that removal of poorly connected metabolites nodes can disrupt subsystems leading to failure of entire networks (Mahadevan and Palsson, 2005; Winterbach et al., 2011). Other studies have attempted to resolve this problem with new network metrics specifically tailored to describe important features of metabolism (Palumbo et al., 2005; Rahman and Schomburg, 2006; Wunderlich and Mirny, 2006; Cooper and Barahona, 2010; Kim et al., 2019; Yeganeh et al., 2020).

**Figure 1:**
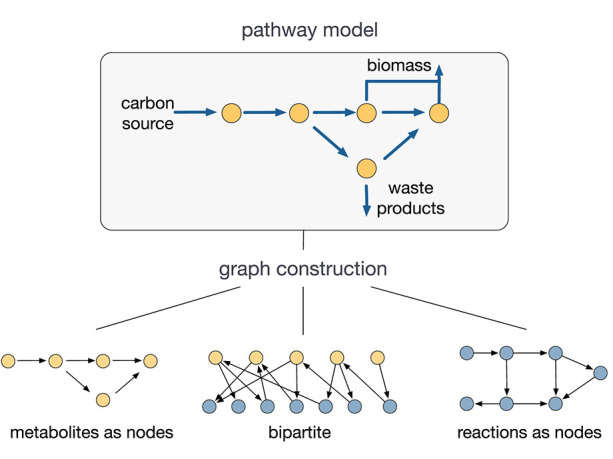
Graph constructions for metabolic networks. Depending on how nodes and edges are defined, several graphs can be built from a single metabolic model (Palsson, 2015). The conclusions drawn from graph analyses depend strongly on the choice of graph. The lack of consensus on the construction of such graphs is a key challenge for the use of network science in metabolic modeling.

A key challenge for the use of network science in metabolic modeling is the lack of consensus on how to build a graph from a metabolic model. For a network with *q* nodes, the graph is encoded through the *q* × *q* adjacency matrix **A**, which has an entry *A*_*ij*_ ≠ 0 if nodes *i* and *j* are connected, and *A*_*ij*_ = 0 otherwise. As illustrated in Figure 1, depending on how nodes and edges are defined, one can build different graphs for the same metabolic model described by the stoichiometry matrix **S** in (2). From a metabolite-centric perspective one can build a graph where the nodes are metabolites and the edges corresponds to reactions between them (Ma and Zeng, 2003b; Asgari et al., 2013). In this case the adjacency matrix is

(3) $$\begin{array}{l} {\text{A}}_{n\times n}=\hat{\text{S}}{\hat{\text{S}}}^{T}, \end{array}$$

where $\hat{\text{S}}$ is the binary version of the stoichiometry matrix **S** (i.e., Ŝ_*ij*_ = 1 when *S*_*ij*_ ≠ 0, and Ŝ_*ij*_ = 0 otherwise). Conversely, from a reaction-centric perspective we can construct graphs with reactions as nodes and edges describing the sharing of metabolites as reactants or products (Ma et al., 2004; Beguerisse-D́ıaz et al., 2018). Such graph has an adjacency matrix

(4) $$\begin{array}{l} {\text{A}}_{m\times m}={\hat{\text{S}}}^{T}\hat{\text{S}}. \end{array}$$

One can also build bipartite graphs, where both metabolites and reactions are nodes of different types (Holme, 2009; Beber et al., 2012), or even hypergraphs where an edge connects a set of reactants to a set of products (Cottret et al., 2010; Pearcy et al., 2016). In addition, all of these graphs can be directed/undirected (when the matrix **A** is asymmetric/symmetric), or weighted/unweighted (where the elements *A*_*ij*_ can have weights encoding different properties). Such modeling choices can strongly influence the conclusions drawn from network analyses (Klamt et al., 2009; Bernal and Daza, 2011; Beguerisse-D́ıaz et al., 2018). For example, the existence of power law degree distributions (Jeong et al., 2000) and the small-world property in metabolism (Wagner and Fell, 2001), two cornerstone concepts in network science, have been disputed (Arita, 2004; Lima-Mendez and van Helden, 2009) and attributed to specific ways of constructing the network graph (Montañez et al., 2010; Bernal and Daza, 2011).

A further limitation of graph-based analyses is their *ad hoc* treatment of pool metabolites, e.g., H_2_O, ATP, NADH, and other enzymatic co-factors. Because pool metabolites participate in a large number of reactions, they can distort and dominate the topological properties of reaction-centric graphs (Ma and Zeng, 2003b). A common approach to mitigate this problem is pruning the pool metabolites from the graph; yet there are no established best practices on how to choose which pool metabolites to prune, or how to mitigate the potential loss of information in so doing (Ma and Zeng, 2003a; Gerlee et al., 2009).

Another challenge arises from the reversibility of metabolic reactions in the graph. Although all biochemical reactions are reversible, they take one direction depending on the physiological conditions. The analysis of reaction-centric graphs typically prescribe a direction for reaction flux, or they split them into forward and backward components (Wagner and Fell, 2001; Helden et al., 2002). Neither of these approaches is ideal: assigning the direction of a reaction based on one condition may not generalize across other conditions, whereas incorporating bi-directional edges increases the complexity of the analysis.

## 4. Flux-Weighted Graphs: Integration of FBA and Network Science

**Figure 2:**
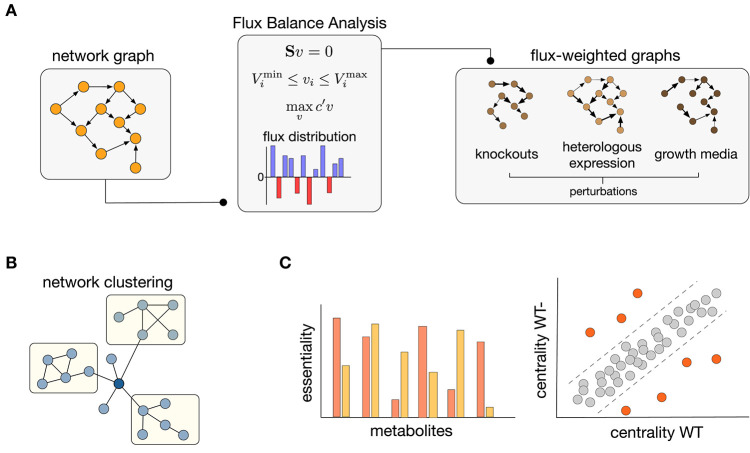
Integration of Flux Balance Analysis and graph theory. **(A)** Construction of flux-weighted graphs. Starting from a network graph, FBA solutions can be used to weight the graph edges, leading to different instances of the same graph for various perturbations such as gene knockouts, heterologous gene expression, or changes in growth conditions. **(B)** Clustering of flux-weighted graphs can reveal hidden groupings in the structure of metabolic networks (Yoon et al., 2007; Beguerisse-D́ıaz et al., 2018). **(C)** Essentiality and node centrality are two areas where flux-weighted graphs offer promising potential. (Left) New essentiality scores can be defined to rank metabolites according to their impact on the phenotype (Koschützki et al., 2010; Riemer et al., 2013; Laniau et al., 2017). (Right) Changes in node centrality between wild type (WT) and deletion (WT-) networks can reveal the molecular players associated to disease phenotypes; orange nodes denote reactions that undergo substantial changes in centrality upon gene deletions (Li et al., 2013; Beguerisse-D́ıaz et al., 2018).

As discussed in previous sections, both FBA and network science require modeling choices that can shape the conclusions drawn from their analyses. Tools from network science have already been employed to improve FBA pipelines in various ways (Lewis et al., 2012). Here we argue that the converse, i.e., using FBA to enrich the metabolic graphs, offers promising avenues to overcome some of their individual shortcomings. Flux information obtained from FBA solutions can be employed to assign direction and strength to the interactions between nodes in a graph. Such flux-weighted graphs allow to constrain their connectivity to various growth conditions, resulting in graphs that do not represent one universal network but are rather tailored to specific environmental or physiological contexts. As illustrated in Figure 2A, the integration of FBA and graph construction can thus result in a highly flexible pipeline to study metabolic connectivity in different functional states of an organism.

Although the literature on this subject is still scarce, a number of studies have demonstrated the potential of the integration of FBA into graph analyses. These studies cover a range of methodologies and applications, including e.g., the identification of biomarkers (Li et al., 2013), detection of metabolic drug targets (Li et al., 2010), and quantification of metabolite essentiality (Riemer et al., 2013; Laniau et al., 2017). In one of the early works in the subject, Smart et al. (2008) proposed an adaptation of FBA that takes into account the connectivity of individual nodes. This idea revealed new insights on how the connectivity of specific metabolites provides robustness to metabolic networks.

Other studies have explored the use of FBA to construct flux-weighted graphs with either metabolites as nodes (Yoon et al., 2007; Koschützki et al., 2010; Riemer et al., 2013) or reactions as nodes (Kelk et al., 2012; Li et al., 2013; Beguerisse-D́ıaz et al., 2018). An alternative approach defined the concept of flux similarity (Li et al., 2010) to build reaction-drug graphs for detection of drug targets in cancer. Most recently, Hari and Lobo (2020) developed Fluxer, a web tool for visualization and analysis of flux-weighted metabolite graphs. The software allows the inclusion of customizable edge weights based on reaction fluxes and can perform multi-reaction knockout simulations.

In terms of applications, most studies have focused on flux-weighted graphs for the analysis of metabolic modularity and essentiality. Next we briefly discuss some of the approaches so far in these two application domains.

### 4.1. Network Clustering

A promising application of flux-weighted graphs is the detection of modular subunits within genome-scale metabolic models (Figure 2B). The idea is that flux-weighted graphs can encode information on the strengths on interactions between graph nodes that are specific to a particular physiological state, as modeled by the FBA solution. This can potentially reveal hidden groupings within metabolism, or how known groupings change across different contexts. For example, Yoon et al. (2007) employed experimentally determined fluxes to build flux-weighted graphs with metabolites as nodes. Using clustering algorithms on the graphs for energy metabolism of rat liver and adipose tissue formation, the approach revealed changes in cluster membership under different physiological flux distributions.

Another promising approach is the “mass flow graph” proposed by Beguerisse-D́ıaz et al. (2018), which uses FBA solutions to weight the edges of graph with reactions as nodes. In this approach, if reaction *R*_*i*_ produces a metabolite *x*_*k*_ that is consumed by *R*_*j*_, then the weight of the edge between both reactions is

(5) $$\begin{array}{l} w_{ij}=\underset{k}{\sum }\text{(mass flow of}x_{k}\text{ from }R_{i}\text{to }R_{j}\text{)}, \end{array}$$

where the sum acts on all the metabolites that are produced by *R*_*i*_ and consumed by *R*_*j*_. The mass flows in (5) are directly computed from the stoichiometric matrix **S** and a flux vector obtained with FBA. Different mass flow graphs can be then computed for FBA solutions corresponding to specific environmental conditions. Thanks to the flux weighting, mass flow graphs avoid the need to prune pool metabolites, a common limitation of reaction graphs (Gerlee et al., 2009). Although pool metabolites do create many connections between functionally unrelated reactions, in mass flow graphs such connections are weak as a result of the flux weighting. This feature allowed the use of multiscale community detection algorithms to study changes in the modular structure of *E. coli* metabolism in various growth media (Beguerisse-D́ıaz et al., 2018).

### 4.2. Centrality and Essentiality

Flux-graph integration has also provided opportunities to explore centrality scores for quantifying essentiality of reactions and metabolites (Figure 2C). One example of this approach (Li et al., 2013) demonstrated that the combination of PageRank centrality (Newman, 2010) with flux information can help identifying candidate biomarker genes in disease. The use of flux-weighted graphs also allows to compare their connectivity between models that lack specific metabolic genes, e.g., in the case of mutants or genetic deficiencies found in metabolic disorders. For example, PageRank centrality was employed in conjunction with mass flow graphs (Beguerisse-D́ıaz et al., 2018) to study structural changes in hepatocyte metabolism in primary hyperoxaluria type 1, a rare metabolic disease characterized by the lack of the *agt* gene involved in glyoxylate breakdown (Pagliarini et al., 2016). This approach showed that reactions which underwent the highest PageRank changes between healthy and diseased states were directly related to the PH1 phenotype (Figure 2C). Importantly, some of the changes in PageRank centrality did not correlate with changes in flux, providing strong evidence that metabolic graphs can encode information that cannot inferred from FBA alone.

A number of other works have sought to define new, metabolism-specific, centrality scores that can reveal new information on the topology of metabolic networks. For example, Koschützki et al. (2010) built a novel “flux centrality” score for metabolites in networks where only the carbon exchanges are modeled as edges. This metric emphasizes the role that a metabolite plays in biomass formation based on both topology and flux, penalizing the impact of highly connected pool metabolites. Riemer et al. (2013) combined the classic notion of metabolic branch points, i.e., metabolites that are substrates to multiple downstream pathways, with reaction fluxes so as to rank metabolites according to various metrics of essentiality. A similar approach to establish metabolite essentiality was presented by Laniau et al. (2017), where they classify metabolites on the basis of their capacity to influence the activation of a target objective function.

## 5. Discussion

Recent discoveries have led to a renewed interest in the interplay of metabolism with other layers of the cellular machinery (Chubukov et al., 2014; Loftus and Finlay, 2016; Tretter et al., 2016; Reid et al., 2017; Tonn et al., 2020). Due to the complexity and scale of metabolic reaction networks, computational methods are essential to tease apart the influence of metabolic architectures on cellular function. Here we have discussed the complementary roles of Flux Balance analysis and network science in the analysis of metabolism at the genome scale. Although both approaches start from the metabolic stoichiometry, they differ in their mathematical foundations and the type of predictions they produce. FBA predictions can be accurate but their effectiveness requires high quality omics datasets. Network science, in contrast, requires nothing more than the metabolic stoichiometry, yet can lead to misleading predictions depending on how the network graph is built. As a result, so far FBA has led to more successful connections with experimental results than network science.

When used in isolation, both FBA and network science can be insufficient to understand changes in metabolic connectivity triggered by physiological or environmental perturbations. Here we argue that the use of flux-weighted graphs (Figure 2A) allows for a natural integration of FBA and network science, applicable in many subject domains. For example, with the rise of big data in the life sciences, there is a growing interest in using patient metabolic signatures to tailor treatments (O'Day et al., 2018). Computational methods can play a key role in detecting drug targets involved in metabolic activity, and how their targeting can disrupt metabolic connectivity. A particularly promising area is cancer treatment, where there is considerable interest on drugs that target specific metabolic enzymes (Neradil et al., 2012; Nishi et al., 2016). Moreover, novel data-driven approaches based on machine learning can also be integrated with FBA (Zampieri et al., 2019; Kavvas et al., 2020) and network science to extend their capabilities into novel applications.

Another promising application domain is industrial biotechnology (de Lorenzo et al., 2018), where microbial cell factories are engineered for production of commodity chemicals and fine products (Lee et al., 2012b). In this field, FBA is widely employed for strain design, with the goal of finding combinations of genetic interventions that maximize production of a desired metabolite. A recent trend is to increase production with synthetic biology tools and dynamic control of gene expression (Brockman and Prather, 2015; Liu et al., 2018). This approach needs computational methods that capture the dynamic reallocation of metabolic flux (Oyarzún and Stan, 2013). Integrating FBA solutions with network models can provide a versatile tool to identify suitable genetic modifications for microbial strains with increased production.

Further developments at the interface of FBA and network science offer a novel way to explore the impact of perturbations on metabolic connectivity. The flexibility of FBA allows for the modeling of metabolic perturbations of various kinds, including changes in growth conditions, deletion of metabolic genes or the action of enzyme inhibitors, whereas the application of network-theoretical tools can bring a broadened understanding of emergent properties of the overall system. This flexibility offers promising potential to deploy network science tools across a range of questions in basic science, biomedicine, and industrial biotechnology.

## Author Contributions

VD and DO conceived the study. All authors contributed to writing the manuscript.

## Conflict of Interest

The authors declare that the research was conducted in the absence of any commercial or financial relationships that could be construed as a potential conflict of interest. The handling editor declared a past co-authorship with one of the author, DO.
